# Comparison of two swept-source optical biometers in eyes with long axial length

**DOI:** 10.3389/fmed.2025.1762378

**Published:** 2026-01-12

**Authors:** Pedro Tañá-Rivero, Paz Orts-Vila, Pedro Tañá-Sanz, Santiago Tañá-Sanz, Juan Álvarez de Toledo

**Affiliations:** 1 Oftalvist, Alicante, Spain; 2 Oftalvist, Barcelona, Spain

**Keywords:** biometry, cataracts, long axial length, optical, optical coherence tomography

## Abstract

**Purpose:**

To assess the repeatability and agreement of two swept-source optical coherence tomography (SS-OCT)-based biometers when measuring biometric parameters in cataract eyes with a long axial length.

**Design:**

Cross-sectional prospective, comparative study.

**Methods:**

One-hundred and twenty-six eyes of 126 patients with cataracts (LOCS III grade equal or less than 3) and axial lengths >25 mm were measured 3 consecutive times using the Argos and IOLMaster 700 SS-OCT optical biometers. Keratometry [K1 (flat) and K2 (steep)], central corneal thickness (CCT), white-to-white (WTW), anterior-chamber-depth (ACD), lens-thickness (LT), and axial length were measured using both instruments. The repeatability for each device was analysed with the within-subject standard deviation (Sw), coefficient of variability, and coefficient of repeatability, and the agreement between the devices was analysed using a Bland–Altman plot.

**Results:**

The acquisition success rate for both biometers was 100%. For K1 and K2, the Sw values were <0.15D and <0.2D, respectively using the two instruments. For CCT, WTW, ACD and LT, the values were 6.56 μm, 0.05 mm, 0.01 mm and 0.01 mm with the Argos, and 3.54 μm, 0.07 mm, 0.01 mm and 0.01 mm with the IOLMaster 700. The Sw values for axial length were 0.01 and 0.03 mm for the Argos and IOLMaster 700, respectively. There were statistically significant differences between the two SS-OCT biometers for all the parameters evaluated (*p* < 0.001) except WTW (*p* = 0.088). The mean differences for K1, K2, CCT, WTW, ACD, LT and axial length were 0.102D, 0.133D, −14.224 μm, −0.018 mm, 0.108 mm, 0.029 mm, and −0.102 mm, respectively.

**Conclusion:**

Both SS-OCT biometers provide repeatable measurements for the different parameters analysed, however, when compared, it is clear that some of them must be assessed carefully in order to be considered interchangeable.

## Introduction

The development of new optical technologies applied to ocular biometry to obtain accurate measurements of different ocular parameters has made a major contribution to the success of cataract surgery. The good outcomes reported in the literature lead us to conclude that optical biometers based on swept source-optical coherence tomography (SS-OCT) technology are likely to become the gold standard for ocular biometry (1). The purpose of SS-OCT biometry is to accurately measure different ocular parameters (2), the most important being axial length when the intraocular lens (IOL) power is calculated for cataract or refractive lens exchange surgery. The increasing number of parameters considered preoperatively and included in the new formulas available on the market is a complication that surgeons should be aware of when predicting IOL power using different devices (3). The use of this technology in specific circumstances, such as eyes with dense cataracts (4–12), has proved to be more successful than in others, or eyes with long or short axial lengths (7, 13–20). It has been reported that various methods for calculating the axial length using SS-OCT, based on either the group refractive index (IOLMaster 700, Carl Zeiss Meditec, Inc., United States) or on the sum-of-segments (Argos, Alcon Labs., United States), may lead to different axial length measurements (21). It has been suggested that the latter is more precise than the former (single equivalent refractive index) when considering eyes with nonstandard anatomies, such as short and long eyes, as a single equivalent refractive index may underestimate or overestimate axial length values, respectively (13). However, to our knowledge, there are no prospective studies comparing these two biometers in a large sample of eyes with long axial lengths.

The purpose of this clinical study was, therefore, to evaluate the repeatability of two SS-OCT optical biometers based on different methods for calculating axial length, and to assess the agreement of the keratometry (K1: flattest keratometry; K2: steepest keratometry), central corneal thickness (CCT), white-to-white distance (WTW), anterior chamber depth (ACD), lens thickness (LT), and, specifically, axial length measurements, in a large sample of eyes with long axial lengths.

## Materials and methods

This was a cross-sectional prospective, comparative study conducted in two Oftalvist clinical centres (Alicante and Barcelona, Spain). The trial complied with the tenets of the Declaration of Helsinki and all the patients recruited provided written informed consent before participating in the study. The study protocol was approved by the Ethics Committee at Hospital Clínico San Carlos [Madrid (Spain), Number 23/774-O_P] and registered in the public German Clinical Trials Registry prior to the start of the study (Identifier Number: DRKS00033316).

### SS-OCT biometers

The Argos SS-OCT biometer uses a wavelength of 1,060 nm measuring at a rate of 3,000 A-scans/s, and is a sum-of-segments biometer that uses different refractive indices to calculate axial length. Keratometry, with *n* = 1.3375, is measured from the OCT image in conjunction with a 2.2 mm diameter ring made up of 16 light emitting diodes; optical distances are measured using the OCT taking into account the following refractive indices: cornea: *n* = 1.375; aqueous and vitreous humours: *n* = 1.336; and crystalline lens: *n* = 1.410. The IOLMaster 700 SS-OCT biometer uses a wavelength of 1,055 nm measuring at a rate of 2,000 scans/s and calculates axial length on the basis of the group refractive index. Keratometry is measured using telecentric keratometry projecting the 950 nm light source onto three zones of the cornea (1.5, 2.5, and 3.2 mm in a mean corneal radius of 7.9 mm, where *n* = 1.3375).

### Clinical procedures

All the patients included in the study underwent a complete preoperative ophthalmological examination that included the measurement of logMAR corrected distance visual acuity (CDVA), manifest refraction, and both biomicroscopy and dilated fundus examinations. The inclusion criteria were patients aged 50 to 85 years, eyes with cataracts graded using the lens opacities classification system (LOCS) III (22) with a value of ≤3, and an axial length value of >25 mm. The exclusion criteria included patients with ocular trauma, previous ocular surgery, poor fixation, corneal disease (i.e., keratoconus), and any other corneal alteration that may affect the measurement process, eyes with a history of moderate to severe dry eye syndrome, any pathology of the anterior segment that may significantly affect the results (i.e., chronic uveitis, iritis or corneal dystrophy), and/or contact lens wear less than a week prior to the ocular biometry.

The ocular biometry measurements were taken three times, in a random order, using each biometer by the same examiner, and only the right eye of each patient was considered for the study. For each device, once the patients had been positioned comfortably in the forehead and chin rest, they were asked to look at the fixation target and blink just before the measurement. The following parameters were recorded using the two devices: K1, K2, CCT, WTW, ACD, LT, and axial length.

### Statistical analysis and sample size calculation

All the variables in the study, K1, K2, CCT, WTW, ACD, LT, and axial length, were calculated as the mean, standard deviation (SD), and minimum and maximum values. The data was entered into a Microsoft Excel spreadsheet (Microsoft Corp., Redmon, WA, United States). For each device, repeatability, based on a standard adopted by the British Standards Institute and the International Organization for Standardisation (23), was assessed by calculating the within-subject standard deviation (Sw), the coefficient of repeatability (CoR), and the coefficient of variability (CoV). The normality distribution was checked using the Kolmogorov–Smirnov utilising SPSS software (IBM Corp., United States). A paired *t*-test, for normality, or a Wilcoxon signed-rank test, for non-normality, was used to assess possible significant differences between the measurements where a *p*-value of <0.05 was considered statistically significant. The agreement between instruments was analysed with a Bland–Altman plot, showing the average difference, confidence interval of the average difference at 95, and 95% limits of agreement.

To calculate the sample size, we considered that the within-subject variance of the Argos device would be non-inferior to that of the IOLMaster700 for axial length measurements in long eyes. A 2 × 6 replicated design was then used to test whether the Argos within-subject variance (*σ*^2^WT) is actually non-inferior to the IOLMaster 700 within-subject variance (*σ*^2^WC), by testing whether the within-subject variance ratio (*σ*^2^WT/*σ*^2^WC) was less than 10 (H0: *σ*^2^WT/*σ*^2^WC ≥10 versus H1: *σ*^2^WT/*σ*^2^WC <10). With 3 replicate pairs, each patient was measured 6 times. The comparison was made using a one-sided, variance-ratio *F*-test (with the Argos within-subject variance in the numerator), with a Type I error rate (*α*) of 0.025. To detect a within-subject variance ratio (*σ*^2^WT/*σ*^2^WC) of 6.6 with 90% power, a total of 126 eyes (one eye per participant) was required. The sample size was computed using PASS 2023 software, version 23.0.2 (NCSS, LLC, Kaysville, Utah, United States). From a previous study (24), we know that the Sw of axial length measurements were 0.018 mm and 0.007 mm, obtained with the Argos and IOLMaster 700 biometers, respectively. This corresponds to a within-subject variance ratio of 6.6. The non-inferiority variance ratio of 10 is deemed a clinically appropriate limit for this study because 10 times the variance obtained for IOLMaster 700 corresponds to a variability that is below 0.1 mm (i.e., approximately 0.25D).

## Results

One-hundred and twenty-six eyes from 126 patients were recruited and analysed in our study. The mean and standard deviation of LOCS III grade for the study sample was 2.33 ± 0.78, ranging from 1 to 3. The mean and standard deviation of patient age was 65.21 ± 7.86 years, ranging from 50 to 81. No adverse events were reported in any patients during the measurement process and all the measurements were carried out and recorded for subsequent analysis. Specifically, the mean spherical equivalent of the eyes in our sample was −5.56 ± 4.51D, and the preoperative Snellen decimal CDVA was 0.65 ± 0.30. Specifically, the axial length acquisition success rate was 100% for both SS-OCT biometers.

### Repeatability of the different ocular parameters

Table 1 shows the mean values, standard deviation, and ranges for the different ocular parameters obtained in the study using the two optical biometers. This table also indicates the *p*-value to find possible statistically significant differences between the devices for each parameter. According to the Kolmogorov–Smirnov test, all the biometric parameters except for LT and axial length followed a normal distribution pattern (*p* > 0.05). Both the LT and axial length measurements obtained from the Argos and IOLMaster 700 deviated significantly from normality (*p* < 0.05). Therefore, parametric paired-sample t-tests were used for normally distributed variables (K1, K2, ACD, WTW, and CCT), whereas the non-parametric Wilcoxon signed-rank test was applied to LT and axial length. The paired t-tests revealed statistically significant mean differences for K1, K2, CCT and ACD CCT (*p* < 0.001) but not for WTW (*p* = 0.088). The Wilcoxon signed-rank test showed significant differences in LT and axial length (*p* < 0.001).

**Table 1:** Mean ± standard deviation (range) of the different parameters examined for the Argos and IOLMaster 700 SS-OCT biometers.

Parameter	Argos	IOLMaster 700	*p*-value
K1 (D)	42.64 ± 1.51 (38.76–46.23)	42.53 ± 1.53 (38.74–46.81)	<0.001^a^
K2 (D)	43.83 ± 1.62 (39.96–49.13)	43.69 ± 1.62 (39.81–48.38)	<0.001^a^
CCT (μm)	574.41 ± 34.39 (467–642)	561.70 ± 35.64 (467–659)	<0.001^a^
WTW (mm)	12.13 ± 0.39 (10.68–13.03)	12.15 ± 0.39 (10.70–13.10)	0.088
ACD (mm)	3.54 ± 0.34 (2.49–4.19)	3.43 ± 0.32 (2.36–4.14)	<0.001^a^
LT (mm)	4.53 ± 0.40 (3.45–5.52)	4.50 ± 0.39 (3.47–5.38)	<0.001^a^
AL (mm)	26.43 ± 1.54 (25.01–32.32)	26.53 ± 1.59 (25.08–32.58)	<0.001^a^

K, keratometry; CCT, central corneal thickness; WTW, white-to-white distance; ACD, anterior chamber depth; LT, lens thickness; AL, axial length.

a Significant differences <0.05.

Table 2 shows the repeatability analysis for each parameter using the two devices, showing values of Sw, CoV and CoR. For keratometry, K1 and K2, the Sw values were <0.15D and <0.2D, respectively, and the corresponding CoV values were <0.3 and <0.4%, with CoR values of about 0.30 and 0.40D, respectively. For WTW, ACD and LT, the values were also low using both devices, the Sw values being 0.05, 0.01 and 0.01 mm, CoV values <0.5%, and CoR values 0.15, 0.04 and 0.05 mm for the three parameters measured with the Argos. These values were similar for those obtained using the IOLMaster 700 (Sw 0.07, 0.01 and 0.01; CoV from 0.25 to 0.60%; and CoR 0.20, 0.03 and 0.03, respectively). The CCT values were higher, indicating the lowest repeatability among all the parameters (largest Sw value, see Table 2). Finally, the axial length values were again low with Sw, CoV, and CoR values of 0.01, 0.06, and 0.04 for the Argos, and 0.03, 0.12, and 0.08 for the IOLMaster 700, respectively. Axial length is the parameter with the best repeatability in relation to the other parameters analysed, demonstrating both devices’ precision in measuring this distance.

**Table 2:** Repeatability analysis for the different parameters assessed using the Argos and IOLMaster 700 SS-OCT biometers.

	Argos			IOLMaster 700		
Parameter	Sw	CoV (%)	CoR	Sw	CoV (%)	CoR
K1 (D)	0.10	0.25	0.29	0.12	0.31	0.36
K2 (D)	0.11	0.26	0.31	0.15	0.36	0.43
CCT (μm)	6.56	1.20	18.18	3.54	0.63	9.81
WTW (mm)	0.05	0.47	0.15	0.07	0.60	0.20
ACD (mm)	0.01	0.45	0.04	0.01	0.34	0.03
LT (mm)	0.01	0.36	0.04	0.01	0.25	0.03
AL (mm)	0.01	0.06	0.04	0.03	0.12	0.08

K, keratometry; CCT, central corneal thickness; WTW, white-to-white distance; ACD, anterior chamber depth; LT, lens thickness; AL, axial length; Sw, within subject standard deviation; CoV, coefficient of variability; CoR, coefficient of repeatability.

### Agreement between the two SS-OCT biometers

**Figure 1:**
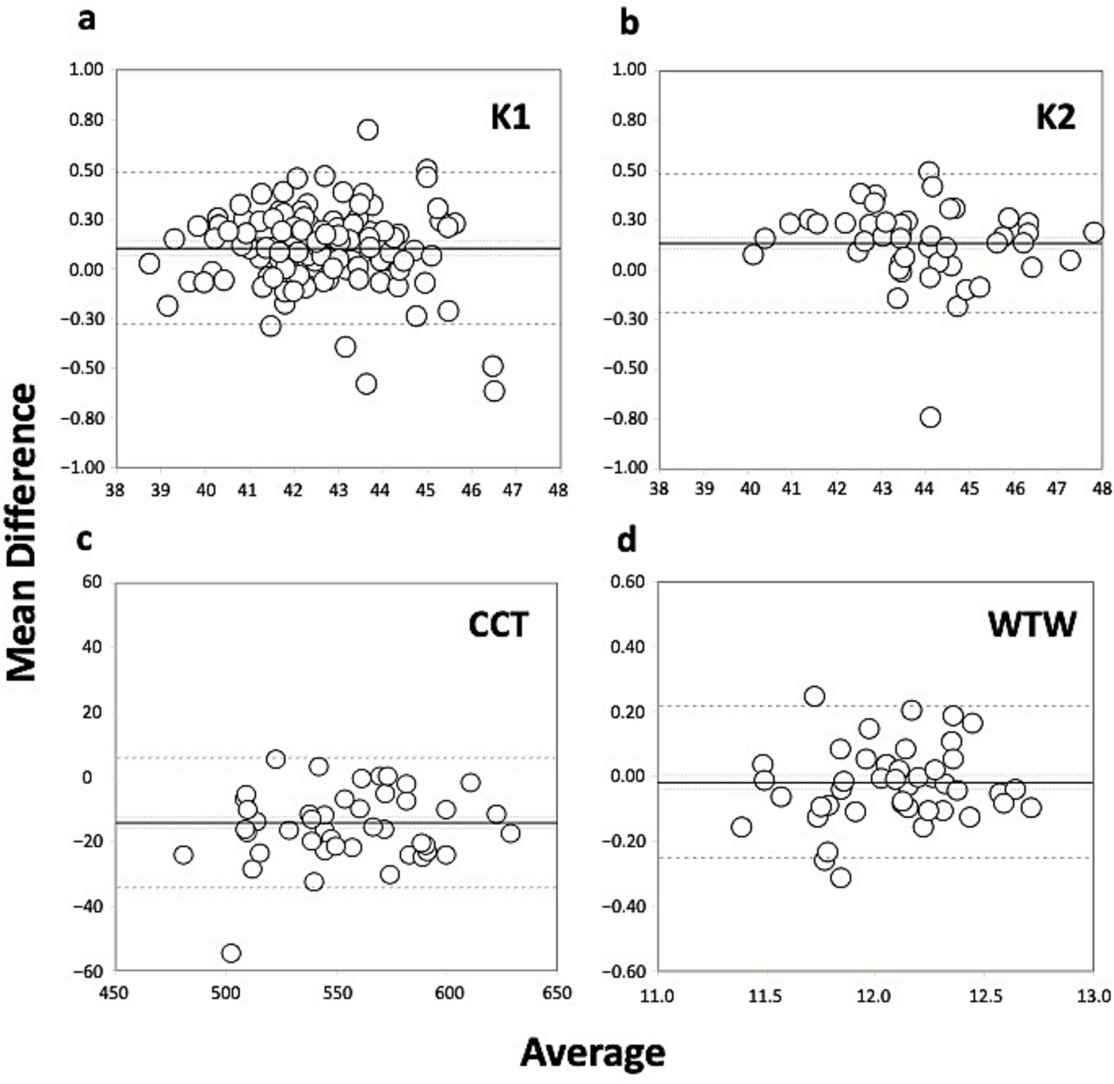
Bland–Altman plots of the mean difference versus the average of K1 (flattest keratometry, D, **a**), K2 (steepest keratometry, D, **b**), CCT (central corneal thickness, microns, **c**), and WTW (white-to-white, mm, **d**) distance used to compare the Argos and IOLMaster 700 SS-OCT biometers. The plots show the mean (continuous line), lower and upper limits of agreement [±1.96 SD (standard deviation), peripheral dotted lines], and the lower and upper confidence intervals (95%).

**Figure 2:**
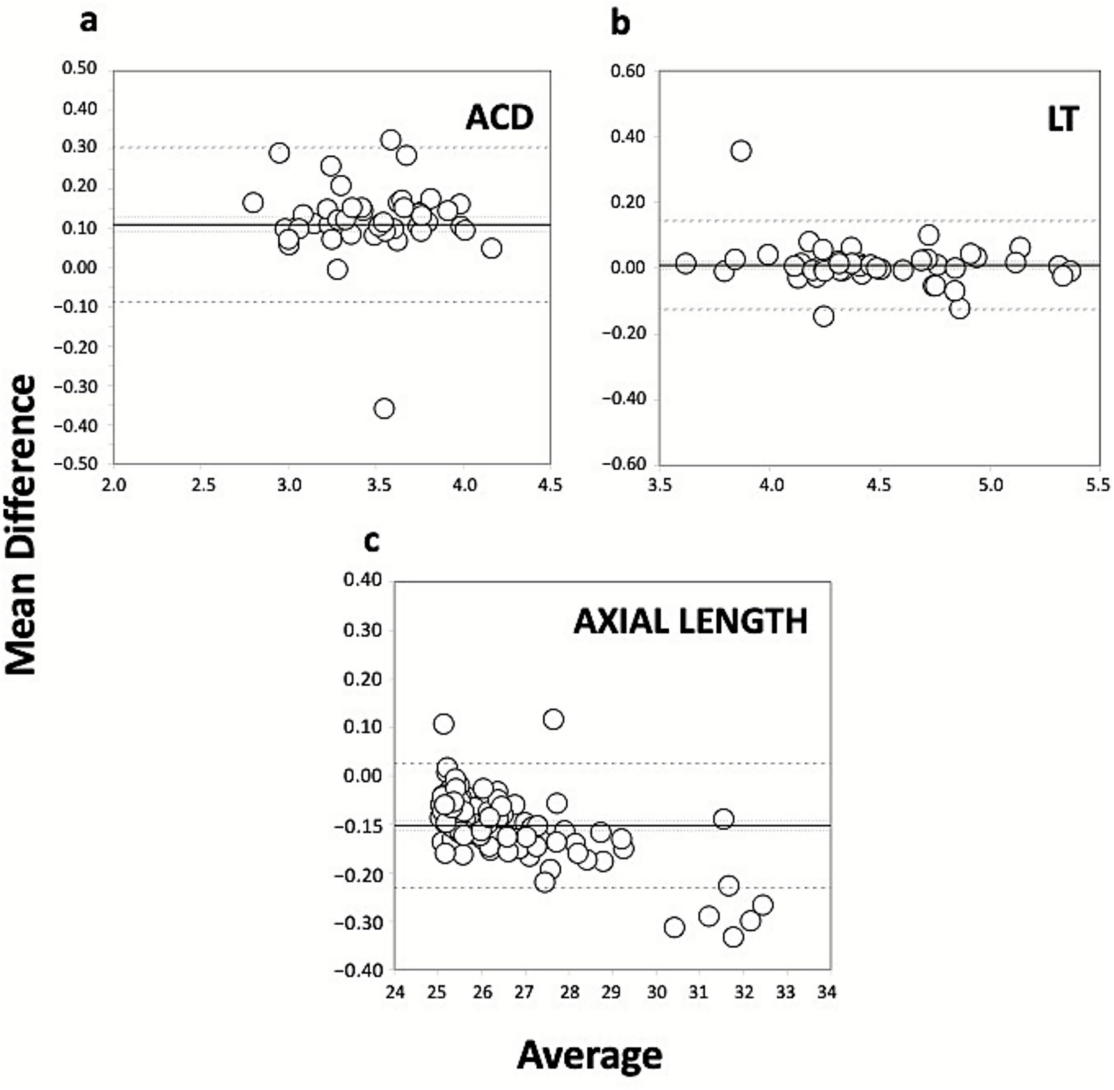
Bland–Altman plots of the mean difference versus the average of ACD (anterior chamber depth, mm, **a**), LT (lens thickness, mm, **b**), and axial length (mm, **c**) used to compare the Argos and IOLMaster 700 SS-OCT biometers. The plots show the mean (continuous line), lower and upper limits of agreement [±1.96 SD (standard deviation), peripheral dotted lines], and the lower and upper confidence intervals (95%).

Table 3 presents the level of agreement between the different ocular parameters obtained using the two SS-OCT biometers, showing the mean difference and standard deviation, 95% confidence interval, 95% limits of agreement, and the limits of agreement (LoA) widths (distance between the upper and lower LoA) for all pairwise comparisons. The Bland–Altman analysis to compare the Argos and IOLMaster 700 SS-OCT biometers was plotted in several graphs shown in Figures 1, 2 for the different ocular parameters. Figure 1 shows the mean difference versus the average for K1 (a), K2 (b), CCT (c), and WTW distance (d); and Figure 2 shows the mean difference versus the average for ACD (a), LT (b), and axial length (c) with the two SS-OCT biometers.

**Table 3:** Agreement between the Argos and IOLMaster 700 SS-OCT biometers for the different parameters examined.

Parameter	Mean difference ± SD	95% CI	95% LoA	LoA width
K1 (D)	0.102 ± 0.194	0.068, 0.136	−0.280, 0.484	0.764
K2 (D)	0.133 ± 0.176	0.102, 0.164	−0.213, 0.479	0.693
CCT (μm)	−14.224 ± 10.282	−16.019, −12.429	−34.377, 5.929	40.306
WTW (mm)	−0.018 ± 0.119	−0.039, 0.002	−0.253, 0.216	0.469
ACD (mm)	0.108 ± 0.099	0.091, 0.126	−0.087, 0.304	0.392
LT (mm)	0.029 ± 0.095	0.013, 0.046	−0.157, 0.216	0.373
AL (mm)	−0.102 ± 0.065	−0.114, −0.091	−0.231, 0.025	0.256

SD, standard deviation; CI, confidence interval; LoA, limits of agreement; K, keratometry; CCT, central corneal thickness; WTW, white-to-white distance; ACD, anterior chamber depth; LT, lens thickness; AL, axial length.

## Discussion

As previously mentioned, optical biometers using SS-OCT technology provide excellent measurements of a number of ocular parameters required for cataract surgery. The aim of this study was to evaluate the repeatability of two of these devices, based on different methods for calculating axial length, and to assess their agreement on different parameters in a specific cohort of eyes with long axial lengths. The results obtained in our study showed good repeatability, with CoV values of less than 2% for all the parameters analysed using the two instruments. Our results agree with those reported by Nemeth and Modis (25), who used the Argos biometer to analyse 40 cataract eyes and reported CoV values of 0.34, 0.32, 1.59, 0.95, 1.35, 0.79, and 0.09%, for K1, K2, CCT, WTW, ACD, LT, and axial length, respectively. Similarly, Huang et al. (26) analysed 109 cataract eyes, also using the IOLMaster 700, and found CoV values of 0.21, 0.46, 1.01, 0.25, 0.13, and 0.07% for Km, CCT, WTW, ACD, LT, and axial length, respectively (first observer). Another study, involving a large sample of 677 cataract eyes measured using the IOLMaster 700, also revealed good outcomes for repeatability with CoV values of 0.50, 0.44, 0.20, 0.41, and 0.01% for CCT, WTW, ACD, LT, and axial length, respectively (27). Note that our study considered only eyes with axial lengths of >25 mm, whereas previous studies considered eyes with different axial lengths [i.e., 21.23 for the 2.5% quantile and 27.89 for the 97.5% quantile (27)]. However, despite this, both devices also performed very well in eyes with long axial lengths. The good repeatability outcomes obtained for both devices give us confidence in their comparison within the agreement sub-study.

If we now focus on the agreement between the two devices, Table 1 shows the mean values obtained using each biometer and Table 3 the mean difference in each parameter evaluated. We found statistically significant differences for all parameters except WTW (*p* = 0.088). For comparison, Figures 1, 2 show the Bland–Altman plots for the different parameters. The agreement of the biometers has been analysed previously in different studies considering eyes with different axial length (7, 11, 14, 28–31). Our study is the first prospective study assessing the two SS-OCT biometers in a large sample of eyes with long axial lengths.

For K1 and K2 the mean differences were 0.102D and 0.133D, respectively (Figures 1a,b). Considering these two values there is no impact on the IOL calculation obtained using one device or another, since the step in IOL manufacturing is higher [note that a difference of 1.0D in the K value would cause a difference of about 1.40D in the IOL power calculation (32)]. Despite this, it should be kept in mind that the LoA slightly exceeds the 0.50D step and may indeed affect the choice of IOL power. In relation to CCT, the mean difference was −14.2 μm with a LoA width of about 40 μm (Figure 1c). The LoA value may impact the intraocular pressure measurement based on the estimation that there is about 1 mm Hg of correction for every 25 μm of deviation (33). The mean difference reported for WTW measurements, which showed no statistically significant difference between the devices, was −0.018 with a LoA width value of about half millimetre which may be clinically significant (Figure 1d). Considering the mean difference for ACD of 0.108 mm and a LoA width of 0.392 mm (Figure 2a), the impact on the IOL power calculation would be not significant [a 1 mm deviation in ACD could lead to a refractive error of 1.5 Din IOL power (32)]. This is also true for LT (Figure 2b), since the mean difference (0.029 mm) and LoA width (0.373 mm) is likely to have no clinical impact on the calculation [0.2 mm variation in LT would change the IOL power by 0.2D using the Olsen or Holladay 2 formulas (34, 35)]. For these two parameters, therefore, the two biometers can be used interchangeably.

Finally, we obtained a mean axial length difference of −0.102 mm (Figure 2c shows the Bland–Altman plot) which yields a refraction error of about 0.27D (34). However, if we consider the LoA width value of 0.256 mm, this surpasses the limits considered clinically negligible (>0.50D) and should be taken into account in the IOL power calculation. Previous retrospective studies have compared the two devices, focusing specially on the axial length and the predictor error/accuracy, and reported certain pertinent information for eyes with long axial lengths. For example, Tamaoki et al. (7) carried out a retrospective comparison of several optical biometers, and specifically assessed the Argos and IOLMaster 700 devices using 622 eyes, 79 of which had an axial length of ≥26 mm. They found that the axial lengths measured by the Argos were significantly shorter than those measured by the IOLMaster 700 (*p* < 0.0001). Using a Bland–Altman analysis they found that the mean difference between the two devices was −0.18 mm (95% LoA: −0.44 to 0.07 mm, 95% CI: −0.21 to −0.15 mm), and this difference showed a statistically significant negative correlation with axial length (*R*^2^ = 0.2613; *p* < 0.0001). This value agrees with the mean difference obtained in our sample (see Tables 1, 3). Tamaoki et al. (7) did not consider this difference clinically negligible and suggested that it may result in erroneous refractive predictions. These authors found a negative correlation between axial length and the lens occupancy ratio, i.e., LT/axial length, and indicated that if this ratio has the highest refractive index change, the measured axial length is also affected. Taking into account that the crystalline lens is estimated to be thinner than their actual thickness, the measured axial length is longer than it is when an equivalent refractive index is used for. They speculated that the difference in measured axial length values between these two biometers depended on the occupancy ratio of the crystalline lens to the axial length. Finally, and after comparing the refractive prediction error, they found no significant differences in this value between the devices; however, they did point out that, due to the principles of axial length using individual refractive indices, the postoperative refractive error of the Argos instrument was about 0.25D myopic in eyes with long axial length. We fully agree with this consideration and it should be kept in mind when calculating IOL power. In another study, Omoto et al. (13) retrospectively compared the same two devices, assessing the accuracy of the IOL power calculation, and concluded that this was clinically acceptable for both instruments. Shammas et al. (15) retrospectively compared axial length measurements based on multiple specific refractive indices for each segment of the eye with those obtained using a single refractive index for the whole eye in a sample of 595 eyes, and reported differences between them in the long eyes (on average, the simulated measurements were longer than the Argos measurements). In another study, Shammas et al. (16) retrospectively analysed the accuracy of newer IOL power formulas in long and short eyes measured using the Argos biometer. They assessed 595 eyes, and specifically 102 long eyes (>24.5 mm). They found that, on average, the segmented axial length was shorter in long eyes than the displayed axial length obtained using a single refractive index for the whole eye. Blehm and Hall (17), in a retrospective study, determined the refractive predictability of the Argos biometer in 55 long eyes (>24.5 mm) after implantation of an extended depth-of-focus IOL and reported 90% of eyes with a ≤0.50D prediction error using the Barrett Universal II formula and 84% using the Barrett True Axial Length formula. Subsequently, in a retrospective study of a larger sample of 92 long eyes (>24.5 mm), Blehm et al. (18) obtained smaller percentages: 82 and 79%, respectively. In their study, Porwolik et al. (19) compared the two biometers and, in a group of only 5 long eyes (>26 mm), found statistically significant differences between them for axial length, this being shorter with the Argos (27.95 versus 28.10 mm, *p* < 0.05). Recently, Rementería-Capelo et al. (20) retrospectively analysed the refractive accuracy of the Argos in 30 eyes with long axial lengths (>26 mm) and 30 eyes with short axial lengths (<22.5 mm) after implantation with the same monofocal IOL (AcrySof IQ, Alcon Labs, Fort Wort, TX, United States) and using the same formulas (Barrett Universal II and Barrett True Axial Length, the latter for comparison based on back calculations). The mean refractive spherical equivalent of the analysed sample was slightly negative for long eyes, while short eyes presented a mean spherical equivalent close to zero. Despite the differences being minimal and postoperative refraction presenting close to emmetropia, the long eyes showed a distribution of postoperative spherical equivalent that trended slightly towards negative refractive values when compared to the short eyes. Blehm and Hall (17) achieved similar refractive outcomes, i.e., the postoperative refraction was slightly more negative for long eyes compared to short eyes. In a previous study, Rementería-Capelo et al. (16) concluded that the Argos biometer showed a good overall refractive accuracy in short and long eyes (>75% of the patients obtained a prediction error within ±0.50D).

In conclusion, the current clinical study analysed the repeatability and agreement of two SS-OCT optical biometers in eyes with cataract and long axial lengths. Our outcomes indicate that both instruments provide good repeatability for different ocular parameters although, when compared, it is clear that some of these should be assessed carefully in order to be considered interchangeable when calculating IOL power.

## Data Availability

The raw data supporting the conclusions of this article will be made available by the authors, without undue reservation.

## References

[1] Montés-Micó R Pastor-Pascual F Ruiz-Mesa R Tañá-Rivero P. Ocular biometry with swept-source optical coherence tomography. J Cataract Refract Surg. (2021) 47:802–14. doi: 10.1097/j.jcrs.0000000000000551, 33315731

[2] Tañá-Sanz P Rodríguez-Carrillo MD Ruiz-Santos M Montés-Micó R Ruiz-Mesa R Tañá-Rivero P. Agreement of predicted intraocular lens power using swept-source optical coherence tomography and partial coherence interferometry. Expert Rev Med Devices. (2021) 18:1219–34. doi: 10.1080/17434440.2021.2008908, 34806515

[3] Tañá-Sanz P Ruiz-Santos M Rodríguez-Carrillo MD Aguilar-Córcoles S Montés-Micó R Tañá-Rivero P. Agreement between intraoperative anterior segment spectral-domain OCT and 2 swept-source OCT biometers. Expert Rev Med Devices. (2021) 18:387–93. doi: 10.1080/17434440.2021.1905518, 33730515

[4] Hirnschall N Varsits R Doeller B Findl O. Enhanced penetration for axial length measurement of eyes with dense cataracts using swept source optical coherence tomography: a consecutive observational study. Ophthalmol Ther. (2018) 7:119–24. doi: 10.1007/s40123-018-0122-1, 29498015 PMC5997603

[5] Henriquez MA Zúñiga R Camino M Camargo J Ruiz-Montenegro K Izquierdo L Jr. Effectiveness and agreement of 3 optical biometers in measuring axial length in the eyes of patients with mature cataracts. J Cataract Refract Surg. (2020) 46:1222–8. doi: 10.1097/j.jcrs.0000000000000237, 32379086

[6] Vasavada SA Patel P Vaishnav VR Ashena Z Srivastava S Vasavada V . Comparison of optical low-coherence reflectometry and swept source OCT-based biometry devices in dense cataracts. J Refract Surg. (2020) 36:557–64. doi: 10.3928/1081597X-20200612-03, 32785730

[7] Tamaoki A Kojima T Hasegawa A Yamamoto M Kaga T Tanaka K . Clinical evaluation of a new swept-source optical coherence biometer that uses individual refractive indices to measure axial length in cataract patients. Ophthalmic Res. (2019) 62:11–23. doi: 10.1159/000496690, 30889604

[8] Tamaoki A Kojima T Hasegawa A Yamamoto M Kaga T Tanaka K . Evaluation of axial length measurement using enhanced retina visualization mode of the swept-source optical coherence tomography biometer in dense cataract. Ophthalmic Res. (2021) 64:595–603. doi: 10.1159/000515054, 33550307

[9] González-Godínez S Saucedo-Urdapilleta R Mayorquín-Ruiz M Velasco-Barona C Moragrega-Adame E Domínguez-Varela IA . Ocular biometry in dense cataracts: comparison of partial-coherence interferometry, swept-source optical coherence tomography and immersion ultrasound. Indian J Ophthalmol. (2022) 70:107–11. doi: 10.4103/ijo.IJO_854_21, 34937218 PMC8917608

[10] Tañá-Rivero P Tañá-Sanz S Pastor-Pascual F Ruiz-Mesa R Montés-Micó R. Axial length measurement failure rates using optical biometry based on swept-source OCT in cataractous eyes. Expert Rev Med Devices. (2022) 19:633–40. doi: 10.1080/17434440.2022.2118047, 36062739

[11] Tañá-Rivero P Aguilar-Córcoles S Tañá-Sanz P Tañá-Sanz S Montés-Micó R. Axial length acquisition success rates and agreement of four optical biometers and one ultrasound biometer in eyes with dense cataracts. Eye Vis. (2023) 10:35. doi: 10.1186/s40662-023-00352-3, 37653460 PMC10472586

[12] Orts-Vila P Tañá-Sanz S Tello-Elordi C Montés-Micó R Tañá-Rivero P. Axial length acquisition success rates and agreement of two swept-source optical biometers in eyes with dense cataracts. Front Med. (2024) 11:1449867. doi: 10.3389/fmed.2024.1449867, 39386744 PMC11461292

[13] Omoto MK Torii H Masui S Ayaki M Tsubota K Negishi K. Ocular biometry and refractive outcomes using two swept-source optical coherence tomography-based biometers with segmental or equivalent refractive indices. Sci Rep. (2019) 9:6557. doi: 10.1038/s41598-019-42968-3, 31024017 PMC6483997

[14] Yang CM Lim DH Kim HJ Chung TY. Comparison of two swept-source optical coherence tomography biometers and a partial coherence interferometer. PLoS One. (2019) 14:e0223114. doi: 10.1371/journal.pone.0223114, 31603903 PMC6788676

[15] Shammas HJ Shammas MC Jivrajka RV Cooke DL Potvin R. Effects on IOL power calculation and expected clinical outcomes of axial length measurements based on multiple vs single refractive indices. Clin Ophthalmol. (2020) 14:1511–9. doi: 10.2147/OPTH.S256851, 32581508 PMC7279716

[16] Shammas HJ Taroni L Pellegrini M Shammas MC Jivrajka RV. Accuracy of newer intraocular lens power formulas in short and long eyes using sum-of-segments biometry. J Cataract Refract Surg. (2022) 48:1113–20. doi: 10.1097/j.jcrs.0000000000000958, 35473887 PMC9514730

[17] Blehm C Hall B. Refractive predictability of a swept source optical coherence tomography biometer in long and short eyes implanted with extended depth of focus intraocular lenses. Clin Ophthalmol. (2023) 17:3525–30. doi: 10.2147/OPTH.S430535, 38026607 PMC10676102

[18] Blehm C Balest Z Blehm AC Hall B. Refractive predictability of two intraocular lens power formulas in long, medium, and short eyes using a swept source optical coherence tomography biometer. Clin Ophthalmol. (2024) 18:2531–7. doi: 10.2147/OPTH.S470158, 39253093 PMC11382796

[19] Porwolik M Porwolik A Mrukwa-Kominek E. Evaluation of selected biometric parameters in cataract patients-a comparison between Argos^®^ and IOLMaster 700^®^: two swept-source optical coherence tomography-based biometers. Medicina. (2024) 60:1057. doi: 10.3390/medicina60071057, 39064485 PMC11278565

[20] Rementería-Capelo LA Contreras I García-Pérez JL Ruiz-Alcocer J. Refractive accuracy of a novel swept-source OCT in patients with short and long eyes. J Ophthalmol. (2025) 2025:9987580. doi: 10.1155/joph/9987580, 39872212 PMC11772054

[21] Savini G Hoffer KJ Carballo L Taroni L Schiano-Lomoriello D. Comparison of different methods to calculate the axial length measured by optical biometry. J Cataract Refract Surg. (2022) 48:685–9. doi: 10.1097/j.jcrs.0000000000000821, 34653096

[22] Chylack LT Jr Wolfe JK Singer DM Leske MC Bullimore MA Bailey IL . The lens opacities classification system III. Arch Ophthalmol. (1993) 111:831–6. doi: 10.1001/archopht.1993.01090060119035 8512486

[23] McAlinden C Khadka J Pesudovs K. Statistical methods for conducting agreement (comparison of clinical tests) and precision (repeatability or reproducibility) studies in optometry and ophthalmology. Ophthalmic Physiol Opt. (2011) 31:330–8. doi: 10.1111/j.1475-1313.2011.00851.x, 21615445

[24] Montés-Micó R. Evaluation of 6 biometers based on different optical technologies. J Cataract Refract Surg. (2022) 48:16–25. doi: 10.1097/j.jcrs.0000000000000690, 34091551 PMC8700306

[25] Nemeth G Modis L Jr. Ocular measurements of a swept-source biometer: repeatability data and comparison with an optical low-coherence interferometry biometer. J Cataract Refract Surg. (2019) 45:789–97. doi: 10.1016/j.jcrs.2018.12.018, 30850124

[26] Huang J Zhao Y Savini G Yu G Yu J Chen Z . Reliability of a new swept-source optical coherence tomography biometer in healthy children, adults, and cataract patients. J Ophthalmol. (2020) 2020:8946364. doi: 10.1155/2020/8946364, 32509343 PMC7246393

[27] Langenbucher A Szentmáry N Cayless A Hoffmann P Wendelstein J Cooke D. Repeatability of biometric measures from the IOLMaster 700 in a cataractous population. PLoS One. (2024) 19:e0297869. doi: 10.1371/journal.pone.0297869, 38330090 PMC10852222

[28] Huang J Chen H Li Y Chen Z Gao R Yu J . Comprehensive comparison of axial length measurement with three swept-source OCT-based biometers and partial coherence interferometry. J Refract Surg. (2019) 35:115–20. doi: 10.3928/1081597X-20190109-01, 30742226

[29] Sabatino F Matarazzo F Findl O Maurino V. Comparative analysis of 2 swept-source optical coherence tomography biometers. J Cataract Refract Surg. (2019) 45:1124–9. doi: 10.1016/j.jcrs.2019.03.020, 31174987

[30] Galzignato A Lupardi E Hoffer KJ Barboni P Schiano-Lomoriello D Savini G. Repeatability of new optical biometer and agreement with 2 validated optical biometers, all based on SS-OCT. J Cataract Refract Surg. (2023) 49:5–10. doi: 10.1097/j.jcrs.0000000000001023, 36026703

[31] Seong J Han SB. Ocular biometry and refractive prediction in short eyes: a comparison of two swept-source optical coherence tomography-based biometers. Bioengineering. (2025) 12:983. doi: 10.3390/bioengineering12090983, 41007228 PMC12467928

[32] Olsen T. Calculation of intraocular lens power: a review. Acta Ophthalmol Scand. (2007) 85:472–85. doi: 10.1111/j.1600-0420.2007.00879.x, 17403024

[33] Kohlhaas M Boehm A Spoerl E Pürsten A Grein H Pillunat L. Effect of central corneal thickness, corneal curvature, and axial length on applanation tonometry. Arch Ophthalmol. (2006) 124:471–6. doi: 10.1001/archopht.124.4.471, 16606871

[34] Olsen T Hoffmann P. C constant: new concept for ray tracing– assisted intraocular lens power calculation. J Cataract Refract Surg. (2014) 40:764–73. doi: 10.1016/j.jcrs.2013.10.037, 24767910

[35] Shammas H Ortiz S Shammas M Kim S Chong C. Biometry measurements using a new large-coherence-length swept-source optical coherence tomographer. J Cataract Refract Surg. (2016) 42:50–61. doi: 10.1016/j.jcrs.2015.07.042, 26948778

